# Editorial: Data assimilation in cardiovascular medicine: Merging experimental measurements with physics-based computational models

**DOI:** 10.3389/fphys.2023.1153861

**Published:** 2023-02-09

**Authors:** E. Lim, Y. Shi, H. L. Leo, A. Al Abed

**Affiliations:** ^1^ University of Malaya, Kuala Lumpur, Malaysia; ^2^ Shaanxi University of Chinese Medicine, Xianyang, Shaanxi, China; ^3^ National University of Singapore, Singapore, Singapore; ^4^ University of New South Wales, Kensington, NSW, Australia

**Keywords:** data assimilation, cardiovascular medicine, computational models, imaging, hemodynamic measurements

## Introduction

Physics-based computational models, constructed based on sound theoretical principles, can give meaningful insight into the complex interrelationship among different parts of the cardiovascular system, such as the heart, vessels and valves. To date, diverse types of physics-based cardiovascular models (Shi et al., 2018; Leong et al., 2019a; Niederer et al., 2019; Ong et al., 2020; Geddes et al., 2022), spanning the disciplines of electrophysiology, electromechanics, solid mechanics, fluid dynamics and cardiovascular reflex, have greatly enhanced our understanding of cardiovascular diseases. However, most of these models used population-based parameters, and thus had significant model uncertainties due to huge intra- and interpatient variability. Rapid advancement of imaging and hemodynamic monitoring technologies has made available useful patient-specific information for personalization of cardiovascular models. Integrating imaging and hemodynamic measurements with physics-based computational models not only enables more accurate prediction of physiological or pathological status for individual patients, but also allows for computation of hemodynamic variables that are challenging to measure experimentally. In addition, patient-specific simulations can provide important insights into the mechanism underlying the disease progression.

This Research Topic focuses on using imaging and hemodynamic measurements for geometry reconstruction, parameterization, or validation of cardiovascular models, with the aim to generate personalized models that could reproduce clinical observations. There is a total of four published articles relevant to basic and clinical studies, covering: 1) deep learning-based framework for feature identification and segmentation; 2) effect of patient-specific boundary conditions or microstructure on biomechanical parameters; and 3) predicting the response of patients to specific therapies using personalized models. We briefly summarize the contributions from the four publications in this Editorial Article in the following.

## Deep learning-based framework for feature identification and segmentation

3D cardiovascular models are typically used to represent anatomically detailed features of the heart and blood vessels (Cuomo et al., 2017; Leong et al., 2019b). Segmentation and reconstruction of 3D geometries based on medical images serves as the first step towards accurate 3D patient-specific hemodynamics analysis. Conventionally, segmentation of the intended structures (e.g., vessel, ventricle) on medical images was performed manually by experienced researchers. However, various challenges were associated with manual segmentation: the need for researchers with rich experience in the interpretation of medical images, highly labor-intensive nature of the manual segmentation process complicated by a large dataset for each patient, interindividual variability in segmentation of complicated anatomy; all of which made patient-specific analyses in large cohorts difficult.

Zhu et al. (**paper 1**) developed a deep-learning-based framework for the identification and segmentation of intracranial aneurysms (IA) using three convolutional neural network (CNN) models. In addition, the impacts of image pre-processing and convolutional neural network architectures on the performance of the network were evaluated. The study dataset consisted of 101 sets of anonymized cranial computed tomography angiography (CTA) images with 140 IA cases. The long-term aim of their work is to predict IA rupture according to the morphological and hemodynamic analysis based on individualized 3D IA models.

## Effect of patient-specific boundary conditions or microstructure on biomechanical parameters

Boundary conditions have a significant impact on biomechanical parameters in a cardiovascular model, such as pressure or velocity field as well as stress or strain distribution (Pirola et al., 2019; Strocchi et al., 2020). Unlike patient-specific geometries, acquisition of *in vivo* pressure and velocity is more challenging as they involve invasive measurements. However, recent advancement of four-dimensional flow MRI data has enabled non-invasive acquisition of detailed flow field in the heart and blood vessels (Pirola et al., 2019). On the other hand, *in-vivo* cardiac Diffusion Tensor Imaging (cDTI) has allowed personalized representation of cardiac microstructure (Ferreira et al., 2014), despite limiting factors such as low resolution, signal-to-noise ratio and spatial coverage.

Compared with using zero pressure or outflow boundary conditions at the abdominal arterial branches of a computational fluid dynamics (CFD) model, the use of patient-specific boundary conditions derived from 4D flow MRI yielded more accurate flow field in the descending aorta of type B aortic dissection cases (**paper 2**). The difference in the hemodynamics among CFD models with different boundary conditions, including flow field, wall pressure, time-averaged wall shear stress and oscillating shear index of the abdominal aorta, is higher in cases where the false lumen involves the abdominal aorta branches. On the other hand, Stimm et al. outlined four interpolation techniques to bridge the gap between the sparse *in-vivo* cDTI data and the biomechanical left ventricle model in generating a 3D representation of the microstructure across the myocardium (**paper 3**). The study showed that errors in fiber representation propagate to the simulation results, where the differences in simulation results (such as strains and ventricular twist) among the four fiber models were correlated with the error introduced by the interpolation model. Reduced interpolation error was correlated with higher material stiffness and more physiological twist.

## Predicting the response of patients to specific therapies using personalized models

Cardiovascular models have been used to investigate aging (Holmes and Lumens, 2018), ventricular diseases (e.g., LV hypertrophy and myocardial infarction) (Tang et al., 2016), valvular diseases (e.g., aortic valve stenosis and regurgitation) (Maragiannis et al., 2015) as well as vascular diseases (e.g., aortic coarctation and aneurysm) (Bäumler et al., 2020). As individual characteristics of patients affect their response to various classes of medications, properly parameterized cardiovascular models based on patient-specific data can be applied to predict the response of a patient to different therapies using reproducible simulation experiments.

Using a modular agent-based model of the cardiovascular and renal systems, Kutumova et al. (**paper 4**) simulated the response of a group of virtual patients with hypertension to antihypertensive therapies with different mechanisms of action. The therapeutic parameters in the model equations simulating the pharmacodynamic effects of different antihypertensive medications were fitted based on published clinical trials results. The model was able to reproduce reasonably the response dynamics following treatment with individual drugs, in accordance with clinical observations.
